# Insulin Regulates Adrenal Steroidogenesis by Stabilizing SF-1 Activity

**DOI:** 10.1038/s41598-018-23298-2

**Published:** 2018-03-22

**Authors:** Ann W. Kinyua, Khanh V. Doan, Dong Joo Yang, My Khanh Q. Huynh, Yun-Hee Choi, Dong Min Shin, Ki Woo Kim

**Affiliations:** 1 Departments of Pharmacology and Global Medical Science, Wonju College of Medicine, Yonsei University, Wonju, 26426 Korea; 2 Department of Oral Biology, BK21 PLUS, Yonsei University College of Dentistry, Seoul, 03722 Korea; 3 Anti-aging Research Institute of BIO-FD&C Co. Ltd., Incheon, 21990 Korea; 4 Present Address: Department of Pharmacology, Tan Tao University, School of Medicine, Tan Tao University Avenue Tan Duc E., City, Duc Hoa, Long An 850000 Vietnam

## Abstract

Development of metabolic syndrome is associated with hyperactivity of the HPA axis characterized by elevated levels of circulating adrenal hormones including cortisol and aldosterone. However, the molecular mechanism leading to the dysregulation of the HPA axis is not well elucidated. In this study, we found that insulin regulates adrenal steroidogenesis by increasing the expression and activity of steroidogenic factor 1 (SF-1) both *in vitro* and *in vivo* and this insulin effect was partly through inhibition of FoxO1. Specifically, insulin increased the protein and RNA levels of SF-1 and steroidogenic target genes. Further, adrenal SF-1 expression was significantly increased by hyperactivation of insulin signaling in mice. Together with the elevated SF-1 expression in adrenal glands, hyperactivation of insulin signaling led to increased aldosterone and corticosterone levels. On the other hand, suppressing the insulin signaling using streptozotocin markedly reduced the expression of adrenal SF-1 in mice. In addition, overexpression of FoxO1 significantly suppressed SF-1 and its steroidogenic target genes implying that the positive effect of insulin on SF-1 activity might be through suppression of FoxO1 in the adrenal gland. Taken together, these results indicate that insulin regulates adrenal steroidogenesis through coordinated control of SF-1 and FoxO1.

## Introduction

The activity of the hypothalamic-pituitary-adrenal (HPA) axis is dysregulated in obese and diabetic patients as well as in different animal models predisposed to metabolic syndrome including *ob/ob* mice and Zucker fatty rat^1–3^. Emerging evidence in the last few decades has shown a close relationship between disorders of the HPA axis and the development of metabolic syndrome as demonstrated by similarities in clinical features of Cushing syndrome and obesity. Cushing syndrome results from the overproduction of adrenocorticotropic hormone (ACTH) by the pituitary glands with the concomitant hypersecretion of cortisol by adrenal glands and is associated with glucose intolerance, hyperlipidemia and hypertension^4^. On the other hand, obesity is associated with an increase in the secreted levels of the adrenal gland hormones including cortisol^5–7^ which is known to increase abdominal fat mass by stimulating the differentiation of preadipocytes to mature adipocytes^8^. The adrenal gland is therefore considered a key player in the development of metabolic syndrome owing to the role of the adrenal hormones in energy and metabolic homeostasis.

Steroidogenic factor 1 (SF-1) is a transcriptional factor belonging to the nuclear receptor superfamily and is predominantly expressed in the adrenal glands, gonads, pituitary gland and ventromedial hypothalamus (VMH). SF-1 plays an important role in steroid hormones synthesis by regulating the transcription of steroidogenic genes including StAR, Cyp11a1, Cyp17, Cyp11b1, Cyp11b2 and 3β-Hsd^9,10^. In addition, SF-1 functions as a metabolic regulator in the brain particularly in the VMH where SF-1 expressing neurons are widespread^11,12^.

The pancreatic hormone insulin is well known for its role in energy metabolism regulation among other physiological functions^13,14^. Previous studies have suggested the involvement of insulin in gonadal steroidogenesis^15,16^. However, the molecular mechanism mediating the insulin effect on steroidogenesis as well as the physiological implication remains to be elucidated. In addition, the role of insulin in regulating the production of adrenal hormones, and the underlying molecular mechanism, has not been clearly demonstrated. In this study we therefore sought to address the role of insulin in steroidogenesis by focusing on adrenal steroidogenesis and metabolic regulation. Our data indicates that insulin regulates adrenal steroidogenesis by up regulating the transcriptional activity of SF-1 both *in vitro* and *in vivo*. Further, we show that overexpression of FoxO1, a downstream transcription factor regulated by insulin, inhibits the expression and transcriptional activity of SF-1 suggesting that insulin might increase the activity of SF-1 by phosphorylating and inactivating FoxO1. Therefore, our findings propose the insulin-FoxO1 pathway as a possible mechanism regulating adrenal hormones production and metabolic homeostasis that is distinct from the canonical cAMP/PKA pathway.

## Results

### Insulin upregulates SF-1 and steroidogenic target genes

**Figure 1:**
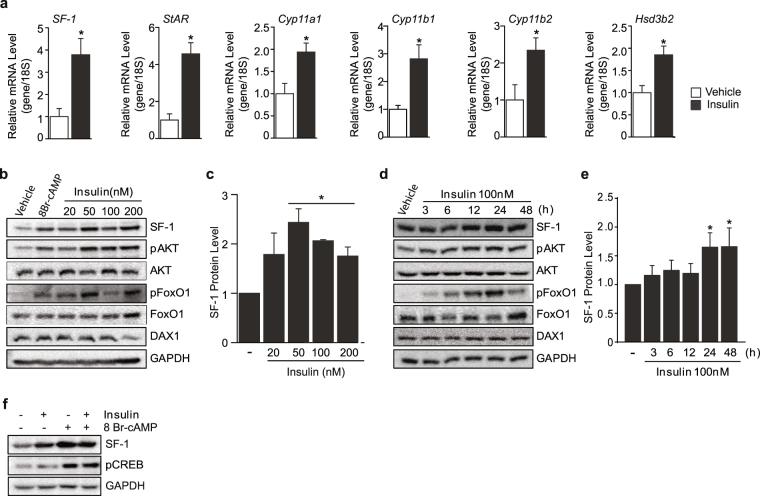
SF-1 and steroidogenic genes are increased by insulin. Relative gene expression of SF-1 and steroidogenic SF-1 target genes after 24 h insulin treatment (**a**). Dose (**b** and **c**) and time (**d** and **e**) dependent effects of insulin on SF-1 and DAX1 protein levels. Note that the activation of insulin signaling was confirmed by phosphorylation of AKT and FoxO1. Effect of insulin and 8Br-cAMP (100 uM) on SF-1 protein levels (**f**). 100 nM of insulin was used for experiments unless otherwise specified. All experiments were performed in triplicates. Original Western blots are provided in the Supplementary information. The values are mean ± SEM (*p < 0.05, Student’s *t*-test).

To investigate the role of insulin in adrenal steroidogenesis, we treated Y1 cells with insulin for 24 h and examined the expression of genes involved in steroidogenesis. Interestingly, insulin markedly up-regulated SF-1 as well as the SF-1 target genes including StAR, Cyp11a1, Cyp11b1, Cyp11b2, and Hsd3b2 all of which are important for adrenal steroidogenic pathway (Fig. 1a). As SF-1 is a known master regulator of steroidogenesis^17^, we monitored the SF-1 protein expression after treating Y1 cells with insulin for 24 h in a dose-dependent manner and also treating 100 nM insulin time dependently. Intriguingly, insulin markedly increased SF-1 protein level dose dependently (Fig. 1b and c). In addition, insulin significantly increased SF-1 time dependently with the strongest effect observed from 24–48 h (Fig. 1d and e). The effect of insulin was accompanied by the phosphorylation of AKT and FoxO1, direct downstream effectors of insulin signaling (Fig. 1b and d). We then tested the effect of insulin on DAX-1 which is a known SF-1 inhibitor^16,18^. We however did not observe any changes on DAX-1 protein by insulin treatment suggesting that the effect of DAX-1 on insulin-mediated SF-1 regulation might be minimal. In addition, treatment of 8Br-cAMP led to an increase in SF-1 protein level accompanied by phosphorylation of CREB while 24 h insulin treatment stimulated the expression of SF-1 in a manner independent of the canonical steroidogenic cAMP/PKA/pCREB pathway^19^ (Fig. 1f). These results highly suggest that insulin might positively regulate the expression of steroidogenic genes by enhancing PKA-independent SF-1 function.

### Insulin stimulates steroidogenic genes expression in SF-1 dependent manner

**Figure 2:**
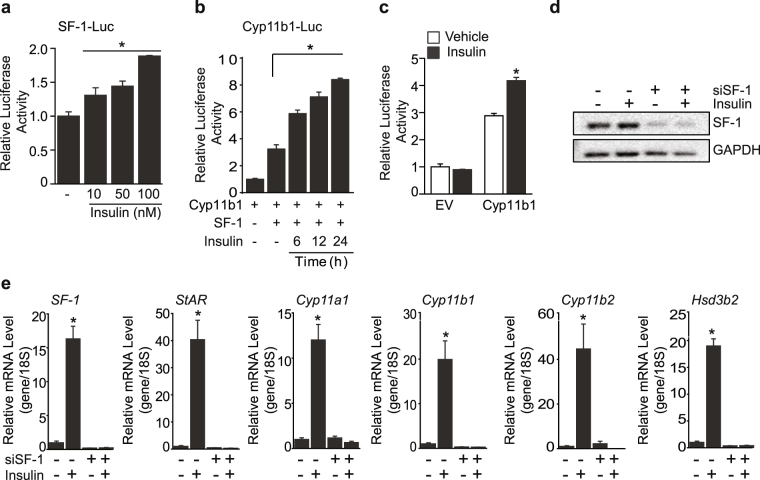
Insulin increases the transcriptional activity of SF-1. Dose-dependent effect of insulin on SF-1 transcription in HEK293 cells (**a**). Increase in Cyp11b1 transcriptional activity by time-dependent insulin treatment after co-transfection of SF-1 (**b**). Relative luciferase activity of Cyp11b1 after insulin treatment in Y1 cells (**c**). Effect of insulin on SF-1 and steroidogenic genes after knocking down SF-1 (**d** and **e**). The values are mean ± SEM (*p < 0.05, Student’s *t*-test, one-way ANOVA). EV, empty vector. SF-1-Luc, SF-1 luciferase construct. Cyp11b1-Lu, Cyp11b1 luciferase construct.

The insulin mediated increase in SF-1 protein and mRNA levels led us to investigate the effect of insulin on the transcriptional activity of SF-1 by performing luciferase assays. Insulin markedly increased SF-1 luciferase activity in a dose-dependent manner (Fig. 2a). Cyp11b1, an enzyme involved in the synthesis of corticosterone was also significantly increased in the presence of SF-1 and insulin (Fig. 2b and Supplementary Fig. S1a). Consistent with the exogenous SF-1 overexpression, Cyp11b1 activation was up-regulated by insulin treatment in Y1 cells in which SF-1 is expressed endogenously (Fig. 2c). To investigate whether the increased expression of steroidogenic genes by insulin was dependent on SF-1, we knocked down SF-1 and then treated insulin (Fig. 2d and e). Up-regulation of steroidogenic genes including StAR, Cyp11a1, Cyp11b1, Cyp11b2, and Hsd3b2 by insulin was markedly blunted by SF-1 knockdown indicating that SF-1 is required for the effect of insulin on adrenal steroidogenic genes (Fig. 2e).

### Insulin-mediated FoxO1 suppression is critical for steroidogenic genes expression

**Figure 3:**
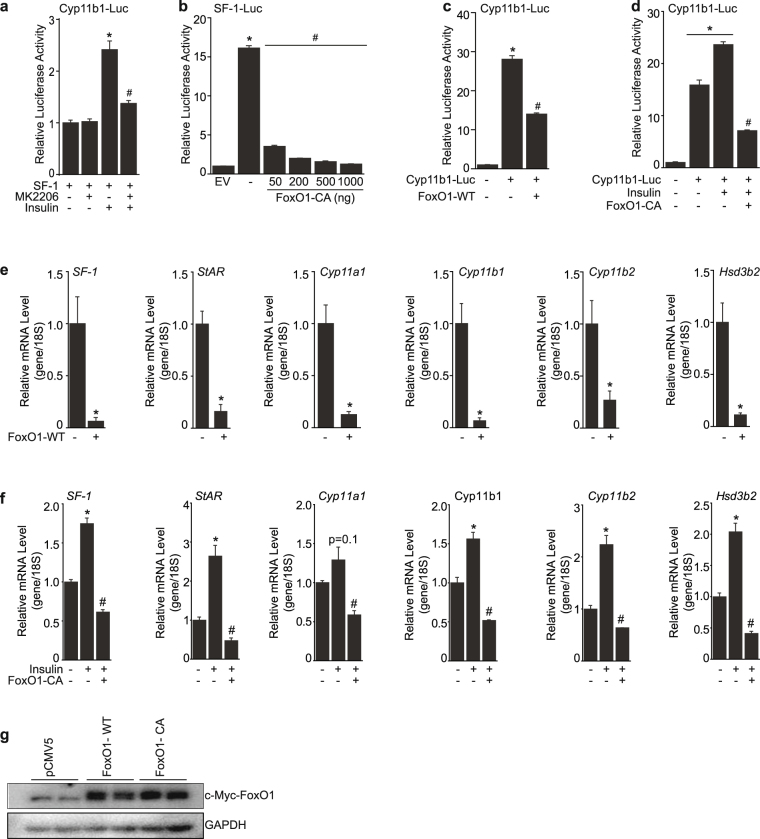
Suppressive effects of FoxO1 on steroidogenic genes expression. Pre-treatment of MK2206 (1 uM) significantly blunted the Cyp11b1 transcriptional activity induced by insulin treatment in HEK 293 cells (**a**). Dose-dependent effect of FoxO1-CA on the SF-1 promoter activity in HEK 293 cells (**b**). Effect of FoxO1-WT on Cyp11b1 promoter activity in Y1 cells (**c**). FoxO1-CA significantly suppressed the Cyp11b1 promoter activity mediated by insulin in Y1 cells (**d**). Overexpression of FoxO1-WT suppressed the expression of steroidogenic genes including SF-1, StAR, Cyp11a1, Cyp11b1, Cyp11b2, and Hsd3b2 in Y1 cells (**e**). Overexpression of FoxO1-CA blunted the insulin-mediated increase of the steroidogenic genes such as SF-1, StAR, Cyp11a1, Cyp11b1, Cyp11b2, and Hsd3b2 in Y1 cells (**f**). Western blot for FoxO1-WT or FoxO1-CA confirming the overexpression experiments in Fig. 3e and f (**g**). The values are mean ± SEM (*p < 0.05 Student’s t-test, #*p* < 0.05 one-way ANOVA).

To gain further molecular insight into how insulin positively regulates steroidogenesis, we pre-treated cells with 1 uM of MK2206, an AKT inhibitor, for 1 h and measured the luciferase activity of Cyp11b1 with or without insulin treatment. The insulin-mediated increase in Cyp11b1 activity was significantly blunted by the AKT inhibitor, implying that the insulin/AKT pathway might be important for regulation of steroidogenic genes activities (Fig. 3a). FoxO1 is phosphorylated by AKT upon activation of insulin signaling and its transcriptional activity is inhibited through cytoplasmic localization by insulin. In addition, previous studies have shown that FoxO1 negatively regulates SF-1 activity in the ventromedial hypothalamus (VMH)^20^. Therefore, we postulated that the upregulation of SF-1 and its steroidogenic target genes by insulin might be as a result of phosphorylation and inactivation of FoxO1 by insulin. To investigate the effect of FoxO1 on SF-1 and steroidogenic target genes, we introduced the constitutively active form of FoxO1 (FoxO1-CA), in which the phosphorylation sites are mutated and is therefore always localized in the nucleus^21^ and examined the luciferase activity of SF-1. FoxO1-CA markedly blunted the transcriptional activity of SF-1 from a low dose of 50 ng (Fig. 3b) and exhibited continuous suppressive effect up to 1 ug. We next overexpressed FoxO1-WT or FoxO1-CA with or without insulin and examined transcriptional activity of Cyp11b1. FoxO1-WT significantly inhibited the promoter activity of Cyp11b1 and further repressive effect was shown by FoxO1-CA (Fig. 3c and d). Additionally, the repressive effect of FoxO1-WT and FoxO1-CA resulted in significant suppression of SF-1 mRNA levels as well as steroidogenic target genes including StAR, Cyp11a1, Cyp11b1, Cyp11b2, and Hsd3b2 (Fig. 3e–g). Taken together, these data highly suggests that FoxO1 negatively regulates the expression of SF-1 and steroidogenic genes and that suppression of FoxO1 by insulin treatment might stimulate steroidogenesis, at least in part, through activation of SF-1.

### Stimulation of adrenal steroidogenesis by activation of insulin signaling

**Figure 4:**
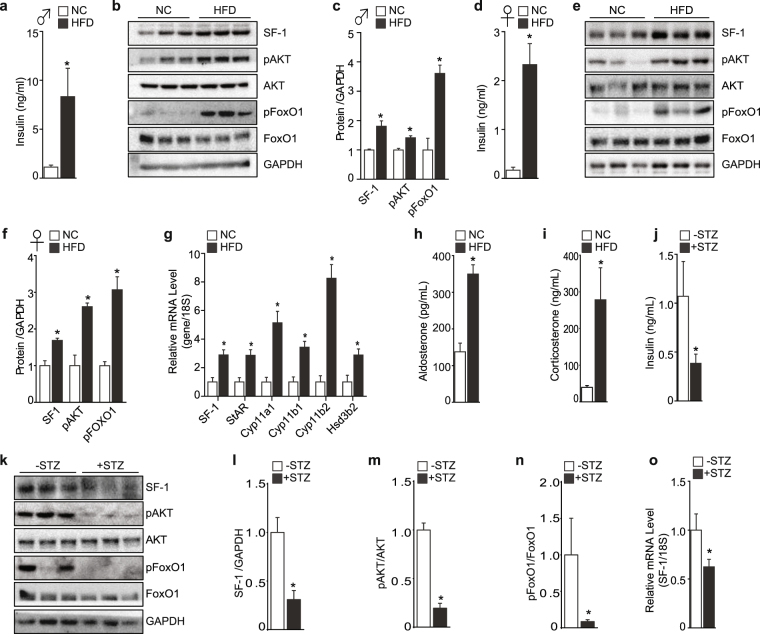
Regulation of adrenal steroidogenesis by insulin. Plasma insulin levels in male mice fed normal chow (NC) or HFD for 8 weeks (n = 4–6) (**a**). Protein levels of indicated proteins from the adrenal gland (**b**). Densitometry of the protein intensity from (**b**) in male mice (**c**). Plasma insulin levels in female mice fed NC or HFD for 8 weeks (n = 4–6) (**d**). Protein levels of indicated proteins from the adrenal gland (**e**). Densitometry of the protein intensity from (**e**) in female mice (**f**). mRNA expression of indicated steroidogenic genes from the adrenal gland (**g**). Plasma aldosterone (**h**) and corticosterone (**i**) levels from male mice fed on NC or HFD. Plasma insulin levels of mice injected with streptozotocin (+STZ, n = 7) and mice injected with sodium citrate as control for vehicle (−STZ, n = 5) (**j**). Protein levels of indicated proteins from the adrenal gland (**k**). Densitometry of the indicated proteins (**l**–**n**). Relative mRNA expression of SF-1 from the adrenal gland (**o**). All the corresponding original Western blot images are provided in the Supplementary information. The values are mean ± SEM (*p < 0.05, Student’s *t*-test).

To confirm our findings that insulin stimulates steroidogenesis and to examine the effect of insulin on adrenal steroidogenesis *in vivo*, we generated a mouse model mimicking increased insulin activity by feeding mice on high fat diet (HFD) for 8 weeks. HFD led to a pronounced increase in plasma insulin levels (Fig. 4a and d) and this was accompanied by a marked hyperactivation of insulin signaling pathway specifically in the adrenal gland, soleus muscle, and liver as shown by the significant increase in phosphorylation of AKT and FoxO1 in both male and female mice (Fig. 4b,c,e and f and Supplementary Fig. S2). The hyperactivation of insulin pathway by short-term HFD exposure clearly showed inactivation of FoxO1 and was accompanied by a significant increase of SF-1 in the adrenal gland (Fig. 4b,c,e and f). Corresponding with the elevated SF-1 protein levels in the adrenal gland, the mRNA levels of steroidogenic genes were also significantly elevated (Fig. 4g and Supplementary Fig. S3). Together with the increase in expression of steroidogenic genes, plasma aldosterone and corticosterone levels were highly elevated in male and female mice (Fig. 4h and i and Supplementary Fig. S3). Next, we generated a mouse model mimicking blunted insulin signaling by injecting mice with streptozotocin (STZ) for 5 days. STZ injection led to a marked reduction in serum insulin levels (Fig. 4j). Importantly, SF-1 protein and mRNA levels in the adrenal gland were markedly reduced in the STZ-injected mice (Fig. 4k–o). The blunted insulin signaling in adrenal gland was confirmed by the decreased levels of pAKT and pFoxO1 (Fig. 4k–n). Therefore, our results highly suggest that insulin positively regulates the expression of SF-1, and the phosphorylation and inactivation of FoxO1 through the insulin signaling might be an underlying mechanism mediating the expression and activity of SF-1.

## Discussion

The synthesis and secretion of adrenal steroid hormones is coordinated by three organs, the hypothalamus, anterior pituitary and adrenal glands referred to as the HPA axis. The hypothalamus secretes corticotrophin releasing hormone (CRH) which stimulates the release of adrenocorticotropic hormone (ACTH) from the pituitary gland. ACTH in turn acts through the melanocortin 2 receptors (MC2R) expressed on the adrenal glands leading to an increase in cAMP and activation of protein kinase A followed by the transcription of steroidogenic genes and subsequent synthesis of steroid hormones^22^. Impaired adrenal steroidogenesis has detrimental effects on glucose and energy homeostasis^4^. On the other hand, dysregulation of metabolic homeostasis is associated with increased secretion of adrenal hormones showing a correlation between dysregulation of HPA axis and metabolic syndrome development^5–7^.

In addition to the canonical steroidogenic pathway, various factors have been shown to contribute to steroid hormones synthesis^23–27^. Although these studies focused on the factors involved in steroidogenesis, the direct molecular mechanisms linking metabolic hormones to adrenal steroidogenesis has not been conclusively shown. As metabolic diseases including diabetes and obesity are associated with dysregulation of adrenal steroids, we postulated that metabolic hormones including insulin might directly influence adrenal steroidogenesis. In the current study, we show that insulin directly regulates adrenal steroidogenesis by activating SF-1 and its steroidogenic target genes through a mechanism that might be independent of the canonical CRH/ACTH/MC2R/PKA pathway. To address the role of insulin on adrenal steroidogenesis, we focused on SF-1 which plays an important role in steroid hormones production as it regulates the genes involved in the steroidogenesis^17^. Insulin treatment markedly increased SF-1 and steroidogenic target genes and this increase was dependent on SF-1 as knockdown of SF-1 failed to upregulate the expression of steroidogenic target genes by insulin (Figs 1 and 2). Together with the previous finding in which deletion of the insulin and insulin-like growth factor 1 receptor was associated with adrenal agenesis^28^, our findings suggests that insulin signaling is not only essential for the adrenal glands development, but also required for adrenal steroidogenesis.

Previous studies have shown that FoxO1 negatively regulates the transcription of ovarian steroidogenic genes including luteinizing hormone β-subunit (LHβ) and the follicle stimulating hormone β (FSH β)^29,30^. In addition, FoxO1 was shown to suppress the expression of SF-1 in the ventromedial hypothalamus (VMH)^20^. This information led to our hypothesis that insulin might regulate the function of FoxO1, a direct downstream effector of insulin signaling, and thereby affect steroidogenic genes in adrenal glands. Therefore, we focused on the role of transcriptional factor FoxO1 in regulation of SF-1 expression and adrenal steroidogenesis. To address the role of FoxO1 on adrenal steroidogenesis, we overexpressed the wild-type form of FoxO1 (FoxO1-WT) and the constitutively active form of FoxO1 (FoxO1-CA) and measured the expression and activity of SF-1 and SF-1 target genes. Overexpression of FoxO1 significantly blunted the expression of SF-1 as well as steroidogenic SF-1 target genes. Similarly, FoxO1-WT and FoxO1-CA blunted the luciferase activity of SF-1 and Cyp11b1 (Fig. 3). Further, overexpression of FoxO1-CA significantly suppressed the elevation of SF-1 and its steroidogenic targets induced by insulin treatment (Fig. 3). These data suggest that the suppressive effect of FoxO1 on SF-1 and steroidogenic genes is blunted by insulin treatment. However, whether the effect of FoxO1 on steroidogenic genes is solely mediated through disinhibition of SF-1 activity requires to be further explored.

To understand the insulin-induced increase in adrenal steroids production observed *in vitro*, we set up a mouse model mimicking hyper-activation of insulin signaling pathway by challenging mice with HFD for 8 weeks. HFD exposure was accompanied by a significant increase in insulin levels and exhibited significant activation of insulin pathway illustrated by marked phosphorylation of AKT and FoxO1 in the adrenal gland. Having confirmed the activation of insulin signaling by HFD exposure, we then monitored the expression of SF-1 and steroidogenic target genes in the adrenal gland. As shown in Fig. 4, SF-1 and other genes involved in steroidogenesis including StAR, Cyp11a1, Cyp11b1, Cyp11b2, and Hsd3b2 were significantly increased (Fig. 4 and Supplementary Fig. S3). In addition, the serum levels of adrenal steroid hormones such as aldosterone and corticosterone were significantly elevated in the HFD fed mice (Fig. 4 and Supplementary Fig. S3). Consistently, low insulin condition induced by STZ administration showed a marked reduction in SF-1 levels (Fig. 4j–o). Therefore, our results highly imply that activation of insulin signaling might be directly involved, at least in part, in the regulation of steroidogenesis in the adrenal gland. In addition, it is important to note that there is possibility that insulin could be indirectly involved in steroidogenesis through insulin-induced hypoglycemia since hypoglycemia activates the HPA axis with the concomitant increase in corticosterone levels^31,32^. Therefore further studies to establish the effect of insulin on the HPA axis would provide further insight into the regulation of steroidogenesis regulated by insulin.

This study shows that FoxO1 negatively regulates adrenal steroidogenesis partly through suppression of SF-1. This finding warrants further studies to investigate the direct effects of FoxO1 on the transcription of steroidogenic genes as well as the interaction between steroidogenic genes and other members of the FoxO family in the adrenal gland. Further, clinical analysis on the expression pattern of FoxO1 and SF-1 in patients with obesity and type 2 diabetes could shed more light on the relationship between HPA axis dysregulation and obesity development.

In summary, our data suggest that insulin contributes to the dysregulation of the HPA axis observed in metabolic syndrome by directly increasing the production of adrenal gland hormones through upregulation of SF-1 and the steroidogenic genes important for steroid hormone synthesis. We suggest that the insulin mediated increase in SF-1 and steroidogenic targets might be a result of phosphorylation and inactivation of FoxO1 through a mechanism that is independent of the canonical steroidogenic cAMP/PKA pathway.

## Materials and Methods

### Materials

Dulbecco’s Modified Eagle Medium (DMEM) was purchased from Hyclone Laboratories Inc. (Logan, UT, USA). Fetal bovine serum (FBS) and penicillin-streptomycin were obtained from Gibco Life Technologies (Carlsbad, CA, USA). Insulin (Cat# I2643) and 8Br-cAMP (Cat# B5386) were purchased from Sigma (St. Louis, MO, USA). MK2206 was obtained from Sellekchem (Houston, TX, USA).

### Cell culture

Y1 cells^33^ were cultured in DMEM enriched with 15% horse serum, 5% FBS and 1% penicillin-streptomycin. HEK 293 cells^34^ were grown in DMEM supplemented with 10% fetal bovine serum (FBS) and 1% penicillin-streptomycin. Both cell lines were maintained at 37 °C under 5% CO_2_. All *in vitro* experiments were performed in triplicates.

### Luciferase assay

Y1 and HEK 293 cells were split in six-well plates at a density of 5 × 10^5^ cells/well in 6-well plates and 3 × 10^5^ cells/well in 12-well plates. The following day, cells were transfected with specific DNAs using Lipofectamine 2000 (Thermo Fisher Scientific, Waltham, MA, USA). Renilla was co-transfected as an internal control. Cells were harvested using luciferase lysis buffer composed of 25 mM Tris Phosphate pH 7.8, 2 mM DTT, 2 mM EDTA, 10% Glycerol and 1% Triton X-100. The relative firefly luciferase and renilla activities were recorded by the BioTek’s Synergy TM 2 machine (BioTek Instruments Inc., Winooski, VT, USA) following injection of luciferase and renilla substrates respectively^34^. The fold change was calculated as the ratio of firefly luciferase and renilla activity normalized to the ratios obtained from control cells transfected with empty vector or treated with vehicle in case of drug treatments. Luciferase substrate (pH8.0) was composed of 200 mM Tris-HCL, 15 mM MgSO_4_, 0.1 mM EDTA, 25 mM DTT, 1 mM ATP, 0.2 mM Coenzyme A and 200 uM Luciferin. Renilla substrate (pH 5.0) was composed of 1.1 M NaCl, 2.2 mM Na_2_EDTA, 0.22 M KH_2_PO4 (pH 5.1), 0.44 mg/ml BSA, 1.3 mM Sodium Azide, 1.43 uM Coelenterazine^35^. The SF-1 luciferase construct is described previously^20^ and the Cyp11b1 luciferase construct was prepared by cloning the promoter region of human Cyp11b1 (−1102 bp) upstream of a luciferase reporter gene in pGL3 basic. FoxO1 wildtype (FoxO1-WT), constitutively active form of FoxO1 (FoxO1-CA) and a control vector pCMV5 used in the overexpression experiments were previously described^20,34^. The SF-1 DNA construct used was generated by cloning full length mouse SF-1 into a pcDNA 3.1 vector as described and validated previously^33,36^.

### Western blot

For protein expression analysis, Y1 cells were treated with the specific compounds, insulin and 8Br-cAMP, time and dose dependently. Cells were harvested through trypsinization and lysed using RIPA buffer composed of 50 mM Tris-HCl (pH 8.0), 150 mM NaCl, 1% Triton X-100, 0.5% sodium deoxycholate and 10% SDS containing protease and phosphatase inhibitor cocktails (Roche, Cat. No. 05892791001 and 04906837001 respectively, Indianapolis, IN, USA). Equal amounts of protein were loaded and separated on a 10% SDS-PAGE gel and the protein levels were detected using enhanced chemiluminescence (ECL) following the standard Western blot procedure. Images were captured by UVP Bio-Spectrum 600 imaging system (Ultra-Violet Products Ltd. Cambridge, UK) after incubation with the antibodies listed below: pAKT (Cat. No. 4060S, 1:10,000), AKT (Cat. No. 2967S, 1:10,000), pFOXO1 (Cat. No. 9461, 1:2,000), FOXO1 (Cat. No. 2880S, 1:2,000) and pCREB (Cat. No.9196S, 1:2,000) from Cell Signaling (Danvers, MA, USA). DAX-1 (Cat. No. SC-841, 1:5,000) and GAPDH (Cat. No. SC-25778, 1:10,000) from Santa Cruz Biotechnology (Santa Cruz, CA, USA). Flag (Cat. No. F1365, 1:2,000) from Sigma (St. Louis, MO, USA). SF-1 protein was detected using rabbit antiserum as described previously^37^. Briefly, full length muse SF-1 cDNA containing an N-terminal 6× -His tag was cloned into a pET-28a(+) vector and transformed overnight using Rosetta (Novagen) competent cells. The cells were harvested and the supernatant decanted for purification. Purified SF-1 protein was obtained by passing the supernatant over a Ni-His bind column and washing with 60 mM imidazole. The purified full length SF-1 protein was injected into rabbits and serum collected. Validation of the SF-1 antibody was conducted by immunohistochemistry analysis of SF-1 expression in wildtype and SF-1 knockout mouse models^38^. All the corresponding original Western blot images are provided in the Supplementary information.

### RNA isolation and quantitative real time PCR

For quantitative real time PCR (Q-PCR) analysis, total RNA was isolated from cell or tissue samples using the Ambion Trizol reagent obtained from Life Technologies (Carlsbad, CA, USA) following the manufacturer’s protocol. cDNA was synthesized from 1 ug of total RNA with the high-capacity cDNA reverse transcription kit purchased from Applied Biosystems (Foster City, CA, USA). Q-PCR was performed using SYBR Green PCR master from Applied Systems using the following mouse primers: 18S; 5′-AACCCGTTGAACCCCATT-3′ and 5′-CCATCCAATCGGTAGTAGCG-3′, SF-1; 5′-CCCTTATCCGGCTGAGAATT-3′ and 5′-CCAGGTCCTCGTCGTACGA-3′, StAR; 5′-GTGGCTGCCGAAGACAATC-3′ and 5′-AGGTGGTTGGCGAACTCTATC-3′, Cyp11a1; 5′-TGAATGACCTGGTGCTTCGT-3′ and 5′-GGCAAAGCTAGCCACCTGTA-3′, Cyp11b1; 5′-GAT ACA GAT CCT GAG GGA GC-3′ and 5′-CCG GCA ACG TCA CAA ACA CA-3′, Cyp11b2; 5′-CAGGGCCAAGAAAACCTACA-3′ and 5′-ACGAGCATTTTGAAGCACCT-3′, Hsd3b2;5′- CCAGGGCATCTCTGTTGTCAT-3′ and 5′-GGTTCTGGGTACCTTTCAGATTGA-3′. 18S was used for normalization of gene expression.

### SF-1 gene silencing

The Y1 mouse adrenocortical tumor cells are known to endogenously express SF-1^33,36,39^. To knockdown SF-1, Y1 cells were transfected with mouse SF-1 siRNA for 48 h using Lipofectamine 2000. The mouse SF-1 siRNA sequence 5′-CAUUACACGUGCACCGAGATT-3, as described previously^39^, was obtained from Cosmo Genetech (Seoul, South Korea). A non-target siRNA sequence 5′UUCUCCGAACGUGUCACGUTT3′ was used as negative control.

### Animal care

All the animal experiments were approved by the Institutional Animal Care and Use Committee (IACUC) of the Yonsei University Wonju College of Medicine. All experiments using mice were performed in accordance with the guidelines and regulations of the IACUC, Yonsei University Wonju College of Medicine. Mice were maintained in a controlled environment with room temperature at 22–23 °C and a 12 hour dark/light cycle. C57BL/6 male and female mice were randomly divided into two experimental groups (n = 4–6). One group was maintained on normal chow (Zeigler, Gardners, PA, USA, Cat. No. Rodent NIH-31 Modified Auto, 4.02 kcal g^−1^) while the other group was fed on high fat diet (Research Diets, New Brunswick, NJ, USA, Cat. No. D12492, 60% fat, 5.24 kcal g^−1^) for 8 weeks. An experimental group (n = 7) mimicking blunted insulin signaling was generated by injecting mice with either 50 mg/kg streptozotocin (STZ) (Sigma, St. Louis, MO, USA) for 5 days or with sodium citrate (control for vehicle, n = 5). Mice were sacrificed after decapitation and tissues collected for further analysis.

### Hormone measurement

For corticosterone and aldosterone measurements, mice were housed in individual cages overnight and blood samples were collected the following day at 2 pm. Corticosterone and aldosterone and levels were measured using ELISA kit obtained from Abcam (ab136933 and ab108821 respectively Cambridge, UK) according to the manufacturer’s instructions. Blood samples for insulin measurement were collected at 10 am following decapitation and insulin levels were measured using ELISA kit obtained from Morinaga Institute of Biological Science (Yokohama, Japan) following the manufacturer’s protocol.

### Statistical analysis

All results are expressed as mean ± SEM following analysis using the GraphPad Prism 5.0 software. Statistical comparisons were made by the Student’s *t*-test or ANOVA and results with a *p*-value < 0.05 were considered to be statistically significant.

## Electronic supplementary material

Supplementary Figure

## References

[1] Guillaume-Gentil C (1990). Abnormal regulation of the hypothalamo-pituitary-adrenal axis in the genetically obese fa/fa rat. Endocrinology.

[2] Plotsky PM, Thrivikraman KV, Watts AG, Hauger RL (1992). Hypothalamic-pituitary-adrenal axis function in the Zucker obese rat. Endocrinology.

[3] Bestetti GE (1990). Changes in the hypothalamo-pituitary-adrenal axis of genetically obese fa/fa rats: a structural, immunocytochemical, and morphometrical study. Endocrinology.

[4] Arnaldi G (2003). Diagnosis and complications of Cushing’s syndrome: a consensus statement. J Clin Endocrinol Metab.

[5] Roberge C (2007). Adrenocortical dysregulation as a major player in insulin resistance and onset of obesity. Am J Physiol Endocrinol Metab.

[6] Pasquali R, Vicennati V, Gambineri A (2002). Adrenal and gonadal function in obesity. J Endocrinol Invest.

[7] Krug AW, Ehrhart-Bornstein M (2008). Adrenocortical dysfunction in obesity and the metabolic syndrome. Horm Metab Res.

[8] Tomlinson JJ, Boudreau A, Wu D, Atlas E, Hache RJ (2006). Modulation of early human preadipocyte differentiation by glucocorticoids. Endocrinology.

[9] Ramayya MS (1997). Steroidogenic factor 1 messenger ribonucleic acid expression in steroidogenic and nonsteroidogenic human tissues: Northern blot and *in situ* hybridization studies. J Clin Endocrinol Metab.

[10] Hanley NA (1999). Expression of steroidogenic factor 1 and Wilms’ tumour 1 during early human gonadal development and sex determination. Mech Dev.

[11] Kinyua AW, Yang DJ, Chang I, Kim KW (2016). Steroidogenic Factor 1 in the Ventromedial Nucleus of the Hypothalamus Regulates Age-Dependent Obesity. PLoS One.

[12] Fujikawa, T. *et al*. SF-1 expression in the hypothalamus is required for beneficial metabolic effects of exercise. *Elife* **5** (2016). 10.7554/eLife.18206 PMC5119890 27874828

[13] Roder PV, Wu B, Liu Y, Han W (2016). Pancreatic regulation of glucose homeostasis. Exp Mol Med.

[14] Roh E, Song DK, Kim MS (2016). Emerging role of the brain in the homeostatic regulation of energy and glucose metabolism. Exp Mol Med.

[15] Devoto L, Christenson LK, McAllister JM, Makrigiannakis A, Strauss JF (1999). Insulin and insulin-like growth factor-I and -II modulate human granulosa-lutein cell steroidogenesis: enhancement of steroidogenic acute regulatory protein (StAR) expression. Mol Hum Reprod.

[16] Ahn SW (2013). Insulin directly regulates steroidogenesis via induction of the orphan nuclear receptor DAX-1 in testicular Leydig cells. J Biol Chem.

[17] Parker KL, Schimmer BP (1997). Steroidogenic factor 1: a key determinant of endocrine development and function. Endocr Rev.

[18] Ito M, Yu R, Jameson JL (1997). DAX-1 inhibits SF-1-mediated transactivation via a carboxy-terminal domain that is deleted in adrenal hypoplasia congenita. Mol Cell Biol.

[19] Aesoy R, Mellgren G, Morohashi K, Lund J (2002). Activation of cAMP-dependent protein kinase increases the protein level of steroidogenic factor-1. Endocrinology.

[20] Kim KW (2012). FOXO1 in the ventromedial hypothalamus regulates energy balance. J Clin Invest.

[21] Van Der Heide LP, Hoekman MF, Smidt MP (2004). The ins and outs of FoxO shuttling: mechanisms of FoxO translocation and transcriptional regulation. Biochem J.

[22] Sewer MB, Waterman MR (2003). ACTH modulation of transcription factors responsible for steroid hydroxylase gene expression in the adrenal cortex. Microsc Res Tech.

[23] Bornstein SR, Rutkowski H, Vrezas I (2004). Cytokines and steroidogenesis. Mol Cell Endocrinol.

[24] Kruse M, Bornstein SR, Uhlmann K, Paeth G, Scherbaum WA (1998). Leptin down-regulates the steroid producing system in the adrenal. Endocr Res.

[25] Giudice LC (1992). Insulin-like growth factors and ovarian follicular development. Endocr Rev.

[26] Zhou P (2013). IGF-I signaling is essential for FSH stimulation of AKT and steroidogenic genes in granulosa cells. Mol Endocrinol.

[27] Park WH, Pak YK (2011). Insulin-dependent suppression of cholesterol 7alpha-hydroxylase is a possible link between glucose and cholesterol metabolisms. Exp Mol Med.

[28] Pitetti JL (2013). Insulin and IGF1 receptors are essential for XX and XY gonadal differentiation and adrenal development in mice. PLoS Genet.

[29] Arriola DJ, Mayo SL, Skarra DV, Benson CA, Thackray VG (2012). FOXO1 transcription factor inhibits luteinizing hormone beta gene expression in pituitary gonadotrope cells. J Biol Chem.

[30] Choi YS (2014). FoxO1 is a negative regulator of FSHbeta gene expression in basal and GnRH-stimulated conditions in female. Endocrinology.

[31] Aizawa T, Yasuda N, Greer MA (1981). Hypoglycemia stimulates ACTH secretion through a direct effect on the basal hypothalamus. Metabolism.

[32] Williams KW (2010). Segregation of acute leptin and insulin effects in distinct populations of arcuate proopiomelanocortin neurons. J Neurosci.

[33] Kim KW (2008). Steroidogenic factor 1 regulates expression of the cannabinoid receptor 1 in the ventromedial hypothalamic nucleus. Mol Endocrinol.

[34] Doan KV (2016). FoxO1 in dopaminergic neurons regulates energy homeostasis and targets tyrosine hydroxylase. Nat Commun.

[35] Hampf M, Gossen M (2006). A protocol for combined Photinus and Renilla luciferase quantification compatible with protein assays. Anal Biochem.

[36] Zhao L (2008). Central nervous system-specific knockout of steroidogenic factor 1 results in increased anxiety-like behavior. Mol Endocrinol.

[37] Kim AC (2008). Targeted disruption of beta-catenin in Sf1-expressing cells impairs development and maintenance of the adrenal cortex. Development.

[38] Kim KW (2011). Steroidogenic factor 1 directs programs regulating diet-induced thermogenesis and leptin action in the ventral medial hypothalamic nucleus. Proc Natl Acad Sci USA.

[39] Baba T (2014). Glycolytic genes are targets of the nuclear receptor Ad4BP/SF-1. Nat Commun.

