# Supplementary material for: Insulin Regulates Adrenal Steroidogenesis by Stabilizing SF-1 Activity
Source: Sci Rep. 2018 Mar 22;8:5025. doi: 10.1038/s41598-018-23298-2 (PMC5864882; doi:10.1038/s41598-018-23298-2)

Insulin Regulates Adrenal Steroidogenesis by Stabilizing SF-1 Activity

Ann W. Kinyua[1], Khanh V. Doan[1,4], Dong Joo Yang[1,2], My Khanh Q. Huynh[1], Yun-Hee Choi[3], Dong Min Shin[2], and Ki Woo Kim[1,2*]

[1]Departments of Pharmacology and Global Medical Science, Wonju College of Medicine, Yonsei University, Wonju, 26426, Korea.

[2]Department of Oral Biology, BK21 PLUS, Yonsei University College of Dentistry, Seoul, 03722, Korea

[3]Anti-aging Research Institute of BIO-FD&C Co. Ltd., Incheon, 21990, Korea.

[4]Present address: Department of Pharmacology, Tan Tao University, School of Medicine, Tan Tao University Avenue Tan Duc E. City, Duc Hoa, Long An, 850000, Vietnam

***Correspondence and requests for reprints, materials should be addressed to:**

Ki Woo Kim (kiwoo@yonsei.ac.kr or iamkiwoo@gmail.com): +82-10-9899-8489 office

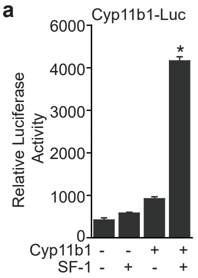

Supplementary Figure S1. Effect of SF-1 on Cyp11b1-luciferase activity (**a**).

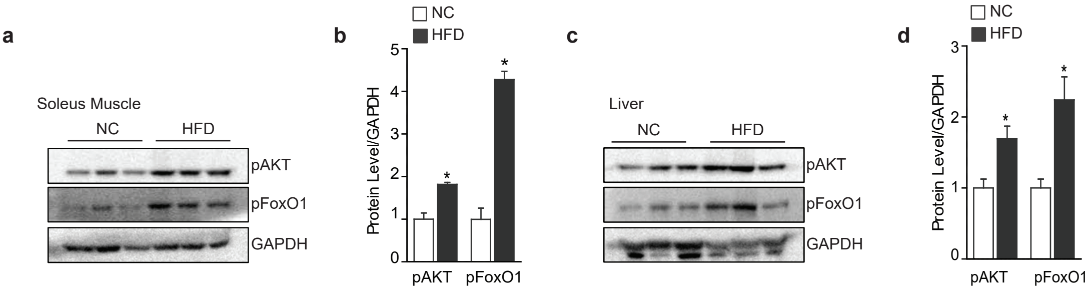

Supplementary Figure S2. Relative phosphorylation levels of AKT and FoxO1 in soleus muscle (**a** and **b**) and liver (**c** and **d**) from male mice. The values are mean ±SEM (*p<0.05, Student's test).

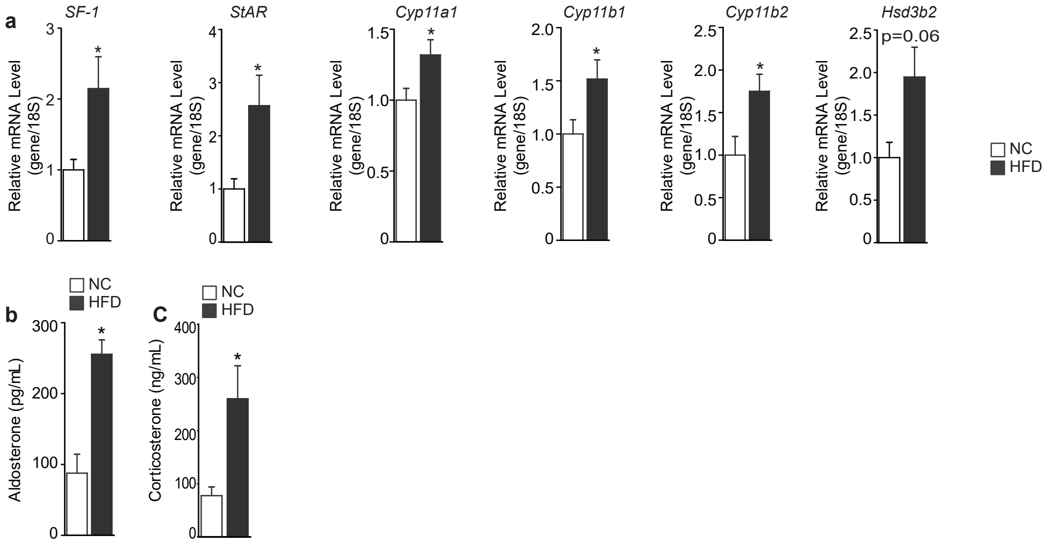

Supplementary Figure S3. mRNA expression of indicated steroidogenic genes from the adrenal gland (**a**). Plasma aldosterone (**b**) and corticosterone (**c**) levels. All samples from female mice. The values are mean ±SEM (*p<0.05, Student's test).

Full-gel blots

Figure 1d

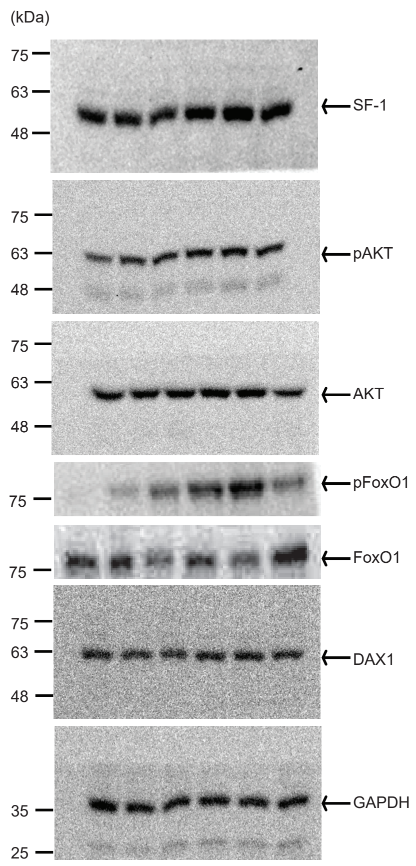

Figure 1b

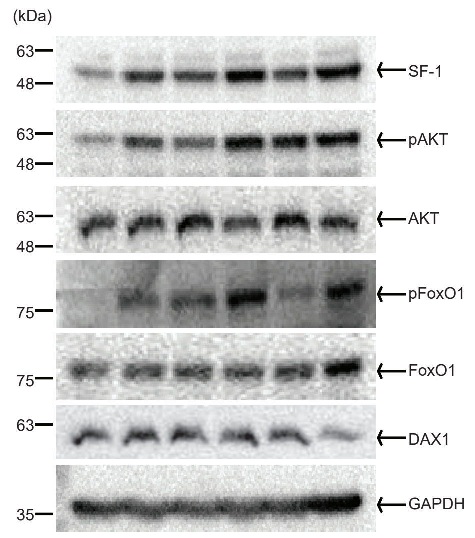

Figure 1f

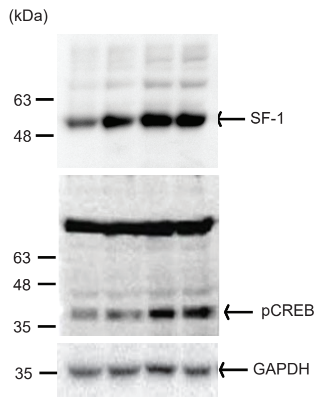

Figure 2d

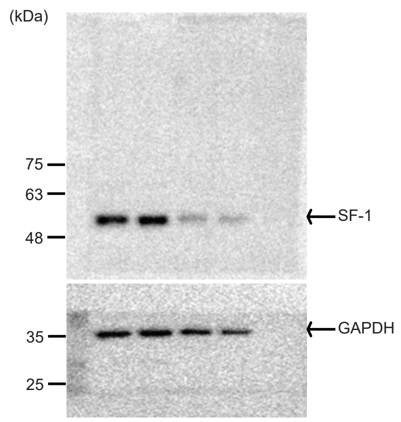

Figure 3g

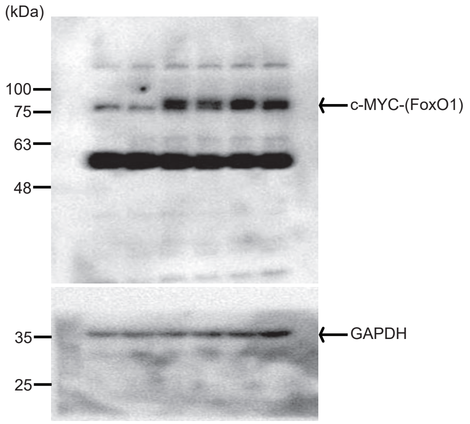

Full gel-blots

Figure 4b

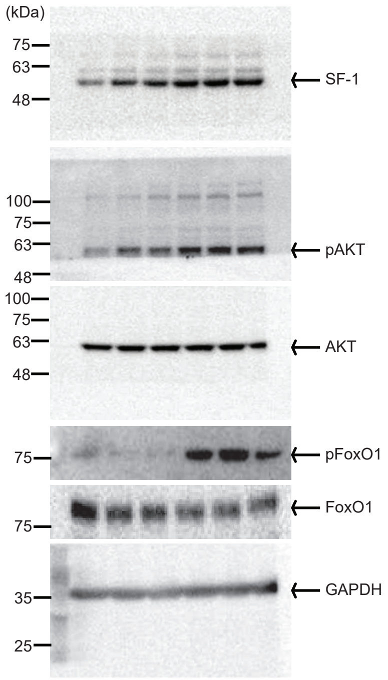

Figure 4e

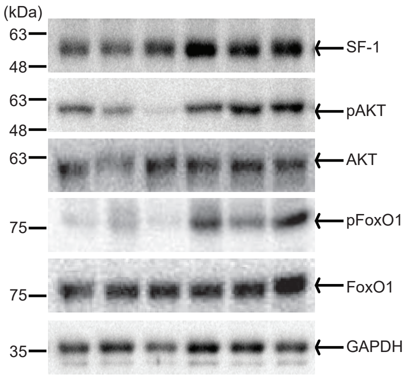

Figure 4k

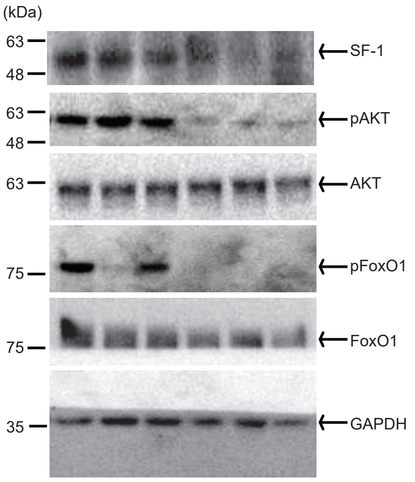

Supplementary Figure S2a

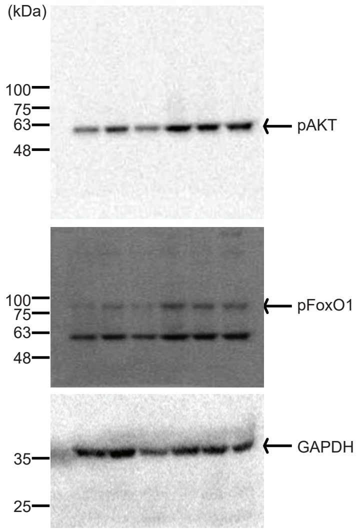

Supplementary Figure S2c

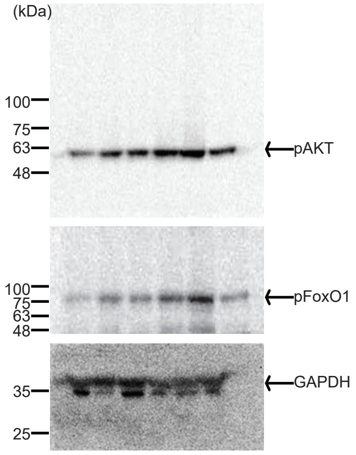

Supplement: Supplementary file 1 — Supplementary Figure [file 41598_2018_23298_MOESM1_ESM.pdf]
